# A Case of Pneumothorax after Treatment with Lenvatinib for Anaplastic Thyroid Cancer with Lung Metastasis

**DOI:** 10.1155/2018/7875929

**Published:** 2018-03-28

**Authors:** Haruhiko Yamazaki, Hiroyuki Iwasaki, Toshinari Yamashita, Tatsuya Yoshida, Nobuyasu Suganuma, Takashi Yamanaka, Katsuhiko Masudo, Hirotaka Nakayama, Kaori Kohagura, Yasushi Rino, Munetaka Masuda

**Affiliations:** ^1^Department of Breast and Endocrine Surgery, Kanagawa Cancer Center, Yokohama, Japan; ^2^Department of Breast and Thyroid Surgery, Yokohama City University Medical Center, Yokohama, Japan; ^3^Department of Surgery, Yokohama City University School of Medicine, Yokohama, Japan

## Abstract

A 63-year-old man was diagnosed with multiple lung metastases from anaplastic thyroid cancer and received lenvatinib. Follow-up computed tomography on day 34 of lenvatinib treatment showed pneumothorax. The pneumothorax was temporarily improved with chest drainage. However, pleurodesis was performed to treat a relapse of the pneumothorax. Pneumothorax during chemotherapy for a malignant tumor is considered a relatively rare complication. This case is the first documentation that pneumothorax may develop during lenvatinib treatment. The possible development of pneumothorax should be considered when lenvatinib is used in patients with lung metastasis.

## 1. Introduction

The incidence of anaplastic thyroid carcinoma (ATC) is 1 to 2% of all thyroid malignancies and is less frequent. However, the prognosis is extremely poor and the 1-year survival rate is reported as 18% [1]. Lenvatinib has a high antitumor effect on thyroid cancer including ATC [2, 3]. In general, pneumothorax during chemotherapy for a malignant tumor is considered a relatively rare complication [4]. Cases of pneumothorax after treatment with lenvatinib have not been reported. Here we report a case of pneumothorax after treatment with lenvatinib for ATC with lung metastasis.

## 2. Case Report

**Figure 1:**
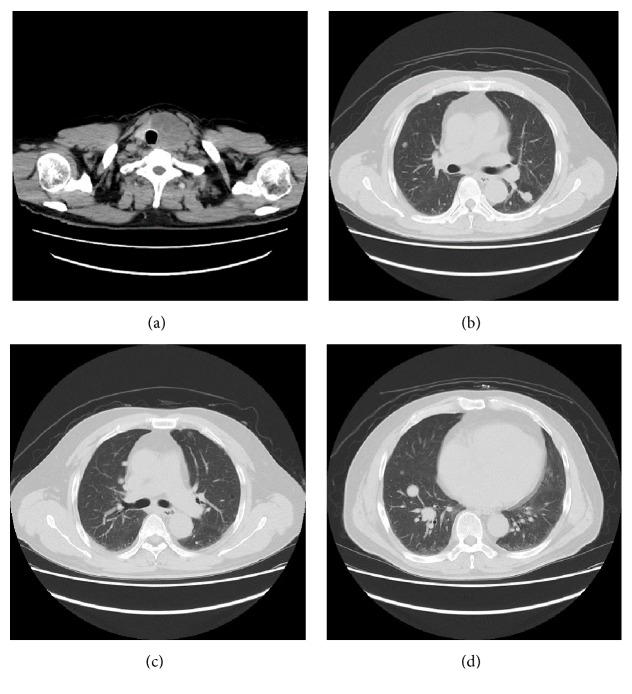
(a–d) Computed tomography showed tumor with eggshell calcification of the thyroid left lobe and multiple lung metastases. There were no bulla and bleb.

**Figure 2:**
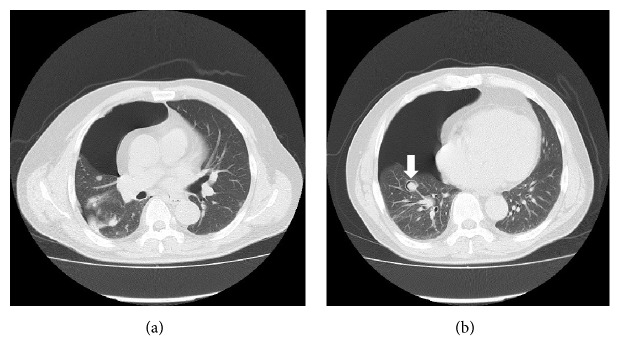
(a, b) Follow-up computed tomography on day 34 of lenvatinib start showed the right pneumothorax and the air was recognized inside a part of lung metastases (arrow).

**Figure 3:**
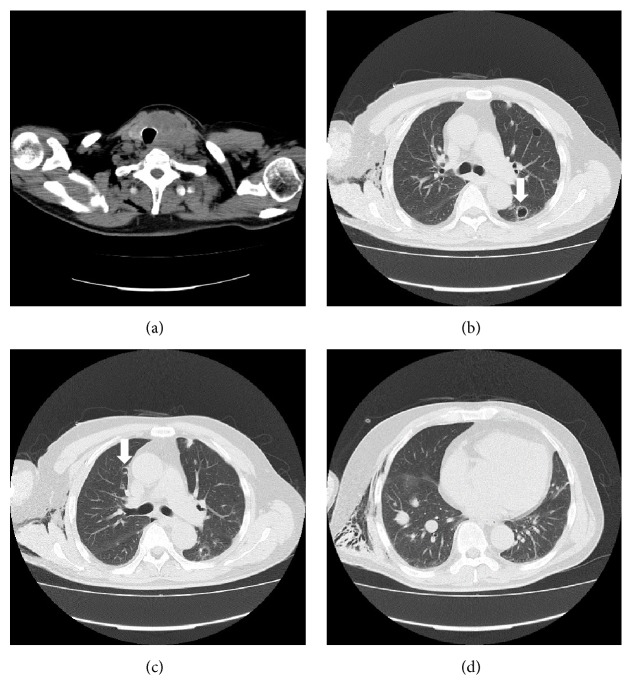
(a–d) Computed tomography on day 73 of lenvatinib start showed that primary tumor was stable disease and there is no recurrence of pneumothorax. Thin wall cavitations were observed in the metastatic lesions (arrow).

A 63-year-old man with a history of treatment for hypertension, hyperuricemia, benign prostatic hyperplasia, sleep apnea syndrome, and bronchial asthma consulted with a nearby doctor regarding hoarseness and mild swallowing difficulty. Computed tomography (CT) findings suggested thyroid cancer with lung metastases, and he visited our hospital (Figure 1). He was hospitalized on the same day as presentation, and a needle biopsy from thyroid tumor resulted in a diagnosis of anaplastic thyroid cancer (ATC). Lenvatinib (24 mg) was started the day after hospitalization. On day 6 of lenvatinib treatment, CT of the primary tumor and lung metastases showed stable disease. He was discharged with no adverse events. On day 20 of lenvatinib administration, the patient developed grade 3 hand–foot syndrome and the lenvatinib was reduced to 20 mg/day. Follow-up CT on day 34 of lenvatinib treatment showed pneumothorax, and he was urgently hospitalized. The patient's height was 174 cm, weight was 79 kg, body temperature was 36.3°C, and blood oxygen saturation was 98% on room air, and he had no breathing difficulty. His blood test results were as follows: thyroid-stimulating hormone, 9.13 *μ*IU/mL; free triiodothyronine, 2.58 pg/mL; and free thyroxine, 1.01 ng/mL. CT showed right pneumothorax, and air was observed inside part of the lung metastases (Figure 2). We established chest drainage, and the pneumothorax improved the next day. However, his swallowing difficulty became exacerbated, and the lenvatinib was increased to 24 mg/day. The pneumothorax was resolved and he was discharged 3 days after hospitalization. At 19 days after discharge, the lenvatinib was reduced to 20 mg/day because of grade 1 pneumothorax and grade 3 proteinuria. Three days later, the pneumothorax became exacerbated and we reestablished chest drainage after emergency hospitalization. Despite the chest drainage, an air leak persisted and the lenvatinib was reduced to 14 mg/day. However, pleurodesis was performed 10 days after starting chest drainage because of continuing air leakage. We removed the chest drain because the pneumothorax had improved the next day. However, the pneumothorax recurred the same day, and we re-started the chest drainage. We removed the chest drain 5 days later, and he was discharged the next day. CT of the primary tumor on day 73 of lenvatinib treatment showed stable disease, and the thin-walled cavitations were observed in the metastatic lesions (Figure 3). The patient developed no further recurrences of pneumothorax.

## 3. Discussion

Lenvatinib is an oral multitargeted tyrosine kinase inhibitor of vascular endothelial growth factors 1, 2, and 3; fibroblast growth factor receptors 1 through 4; platelet-derived growth factor receptor *α*; and the RET and KIT signaling networks. The SELECT trial and a phase II trial conducted in Japan showed that lenvatinib was effective for unresectable thyroid cancer [2, 3]. In the SELECT trial, hypertension was the most frequent adverse event (67.8%), followed by diarrhea (59.4%) and fatigue (59.0%) [2]. However, the occurrence of pneumothorax was not reported. Primary spontaneous pneumothorax is mainly caused by the rupture of a bleb or of a bulla [5]. In this case, however, the CT before the treatment of lenvatinib did not show such lesion. We observed lesions that caused thin wall cavitation by CT after improvement of pneumothorax, which we hypothesise to be the source of the air leak. In general, pneumothorax during chemotherapy for a malignant tumor is considered a relatively rare complication [4]. The reported incidence rate is relatively high in patients with sarcoma (about 2%) [6]. Various causes of pneumothorax during chemotherapy have been surmised. Yamada et al. [7] classified them as follows: rupture of bullae or blebs directly under the pleura during chemotherapy, formation of bronchopleural fistulas secondary to tumor necrosis, development of pleural lesions secondary to damage to the lung parenchyma induced by chemotherapy or radiation therapy, formation of cavities or emphysematous lesions in the peripheral tissues with subsequent rupture by the check-valve mechanism because of obstruction or stenosis of bronchi due to tumors, and elevation of intrathoracic pressure caused by vomiting as a side effect of chemotherapy, resulting in rupture of the pleura. In this case, air was observed inside part of the lung metastases; therefore, the second mechanism (formation of bronchopleural fistulas secondary to tumor necrosis) was considered to be involved in the onset of pneumothorax. Pneumothorax has also been reported in association with other molecular-targeted drugs, including sunitinib for renal cell cancer [8], pazopanib for soft tissue sarcoma [9], and bevacizumab for colon cancer or breast cancer [10, 11]. These patients also had lung metastasis, and the mechanism of onset was similar to our case. In our case, the pneumothorax temporarily improved with chest drainage, but it subsequently recurred and pleurodesis was performed. The pneumothorax recurred again immediately after pleurodesis and finally improved with chest drainage. The series of treatments took 1 month to complete. In some reported cases, however, the treatment lasted several months [7, 11]. Pleurodesis is an indication for patients who cannot or refuse to undergo surgery [12]. Bleeding tendency and delayed wound healing are adverse effects of lenvatinib, so withdrawal of the medication is necessary before surgery. Our patient had a risk of sudden death due to the increase in size of the primary lesion, and long-term withdrawal of lenvatinib was difficult. Therefore, surgery was not considered a treatment option. In some cases, it is necessary to reduce, withdraw, or change the patient's anticancer drugs to allow for treatment of pneumothorax; during that time, however, the primary lesion may progress. The possible onset of pneumothorax must be considered when lenvatinib is used for thyroid cancer in patients with lung metastasis.
